# *catena*-Poly[[diaqua­dipyridine­zinc(II)]-μ-succinato]

**DOI:** 10.1107/S1600536808006764

**Published:** 2008-03-14

**Authors:** Tanwawan Duangthongyou, Sutatip Siripaisarnpipat

**Affiliations:** a Department of Chemistry, Faculty of Science, Kasetsart University, Bangkok 10930, Thailand

## Abstract

In the title compound, [Zn(C_4_H_4_O_4_)(C_5_H_5_N)_2_(H_2_O)_2_]_*n*_, the Zn^II^ ion (site symmetry 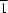) is coordinated in an octahedral geometry by two pyridine mol­ecules, two water mol­ecules and two bridging centrosymmetric *O*-monodentate succinate dianions to create one-dimensional polymeric chains. The chains are cross-linked by O—H⋯O hydrogen bonds, forming sheets.

## Related literature

For a related structure containing fumarate ions, see: Ohmura *et al.* (2003 ▶).
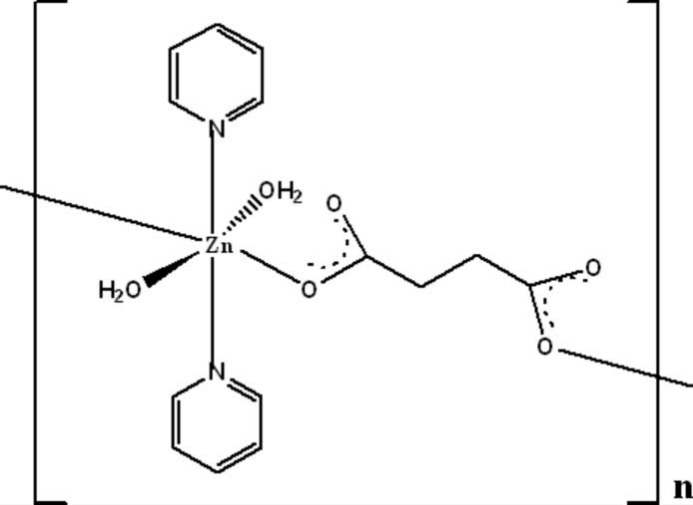


## Experimental

### 

#### Crystal data

[Zn(C_4_H_4_O_4_)(C_5_H_5_N)_2_(H_2_O)_2_]
*M* *_r_* = 375.67
Orthorhombic, 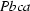

*a* = 11.8142 (8) Å
*b* = 8.9111 (7) Å
*c* = 14.9705 (10) Å
*V* = 1576.06 (19) Å^3^
*Z* = 4
Mo *K*α radiation
μ = 1.59 mm^−1^
*T* = 273 (2) K
0.08 × 0.08 × 0.04 mm

#### Data collection

Bruker–Nonius APEXII CCD diffractometer
Absorption correction: multi-scan (*SADABS*; Sheldrick, 2007 ▶) *T*_min_ = 0.804, *T*_max_ = 0.931
9256 measured reflections
1814 independent reflections
1200 reflections with *I* > 2σ(*I*)
*R*_int_ = 0.067

#### Refinement

*R*[*F* ^2^ > 2σ(*F* ^2^)] = 0.065
*wR*(*F* ^2^) = 0.107
*S* = 1.13
1814 reflections
138 parameters
H atoms treated by a mixture of independent and constrained refinement
Δρ_max_ = 0.55 e Å^−3^
Δρ_min_ = −0.46 e Å^−3^

### 

Data collection: *COLLECT* (Hooft, 1998 ▶); cell refinement: *DENZO* (Otwinowski and Minor, 1997 ▶) and *COLLECT*; data reduction: *DENZO* and *COLLECT*; program(s) used to solve structure: *SHELXS97* (Sheldrick, 2008 ▶); program(s) used to refine structure: *SHELXL97* (Sheldrick, 2008 ▶); molecular graphics: *Mercury* (Macrae *et al.*, 2006 ▶); software used to prepare material for publication: *Mercury* and local program.

## Supplementary Material

Crystal structure: contains datablocks global, I. DOI: 10.1107/S1600536808006764/hb2701sup1.cif

Structure factors: contains datablocks I. DOI: 10.1107/S1600536808006764/hb2701Isup2.hkl

Additional supplementary materials:  crystallographic information; 3D view; checkCIF report

## Figures and Tables

**Table:** 

Zn1—O2	2.064 (3)
Zn1—O3	2.110 (3)
Zn1—N1	2.170 (4)

**Table:** 

O2^i^—Zn1—N1^i^	88.88 (14)

Symmetry code: (i) 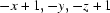.

**Table 2:** Hydrogen-bond geometry (Å, °)

*D*—H⋯*A*	*D*—H	H⋯*A*	*D*⋯*A*	*D*—H⋯*A*
O3—H3*A*⋯O1	0.82	1.94	2.690 (5)	152
O3—H3*B*⋯O1^ii^	0.74 (6)	1.97 (6)	2.687 (5)	164 (5)

Symmetry code: (ii) 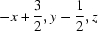.

**Fig. 1.**
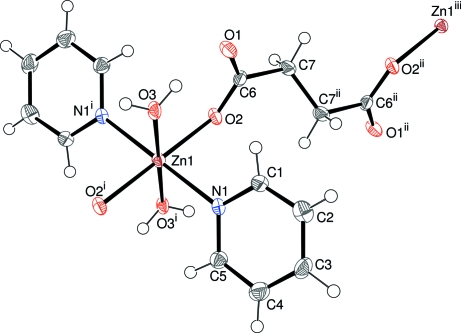
A fragment of the polymeric chain in (I) showing 50% displacement ellipsoids (arbitrary spheres for the H atoms). Symmetry codes: (i) 1 - x, -y, 1 - z; (ii) 1 - x, 1 - y, 1 - z; (iii) x, y + 1, z.

**Fig. 2.**
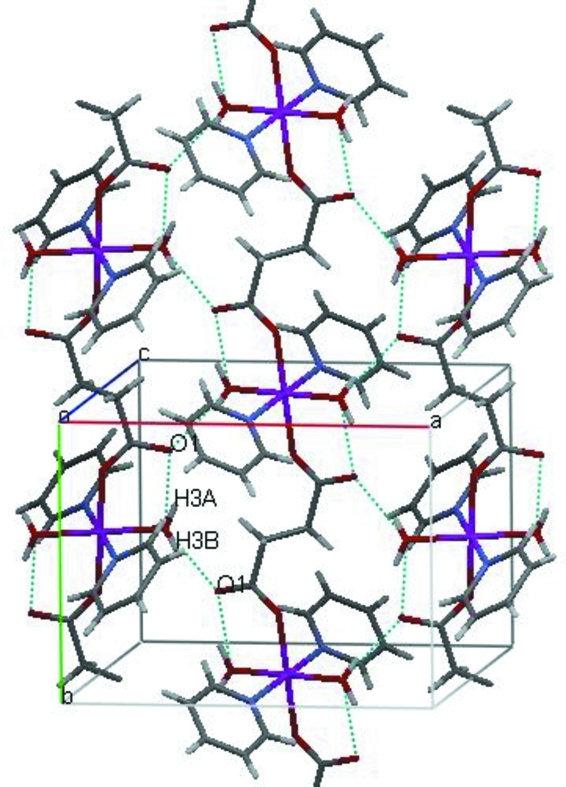
The packing in (I) viewed along the b axis with hydrogen bonds indicated by dashed lines.

## supplementary crystallographic information

### Comment

The molecular structure of the title compound, (I), (Fig. 1), consists of zinc(II) ions linked through succinate bridges to create one-dimensional polymeric chains. Each Zn^II^ ion (site symmetry 1) is further coordinated by two pyridine molecules and two water molecules resulting in a slightly distorted *trans*-ZnN_2_O_4_ octahedral geometry (Table 1).

The coordinated water molecules form both intrachain O—H···O hydrogen bonds with the uncoordinated carboxyl group of a succinate ligand within the chain and intermolecular hydrogen bond with those in an adjacent chain (Fig. 2, Table 2). For a related structure, see Ohmura *et al.* (2003).

### Experimental

A pyridine solution (10 ml) of succinic acid (0.116 g, 1 mmol) was added to an aqueous solution (10 ml) of zinc acetate dihydrate (0.148 g, 0.673 mmol). The mixture was then allowed to stand for several days at room temperature, after which colourless blocks of (I) precipitated.

### Refinement

All the H atoms except H3A were located in a difference map and their positions and *U*_iso_ values were freely refined. H3A was geometrically placed (O—H = 0.82 Å) and refined as riding with *U*_iso_(H) = 1.5 *U*_eq_(H).

### Crystal data

**Table:**

[Zn(C_4_H_4_O_4_)(C_5_H_5_N)_2_(H_2_O)_2_]	*F*_000_ = 776
*M**_r_* = 375.67	*D*_x_ = 1.583 Mg m^−^^3^
Orthorhombic, *P**b**c**a*	Mo *K*α radiation λ = 0.71073 Å
Hall symbol: -P 2ac 2ab	Cell parameters from 2052 reflections
*a* = 11.8142 (8) Å	θ = 2.9–27.5º
*b* = 8.9111 (7) Å	µ = 1.59 mm^−^^1^
*c* = 14.9705 (10) Å	*T* = 273 (2) K
*V* = 1576.06 (19) Å^3^	Block, colourless
*Z* = 4	0.08 × 0.08 × 0.04 mm

### Data collection

**Table:**

Bruker–Nonious APEXII CCD camera on κ-goniostat diffractometer	1814 independent reflections
Radiation source: fine-focus sealed tube	1200 reflections with *I* > 2σ(*I*)
Monochromator: graphite	*R*_int_ = 0.067
Detector resolution: 4096x4096pixels / 62x62mm pixels mm^-1^	θ_max_ = 27.6º
*T* = 273(2) K	θ_min_ = 3.2º
φ and ω scans	*h* = −15→15
Absorption correction: multi-scan(SADABS; Sheldrick, 2007)	*k* = −11→10
*T*_min_ = 0.804, *T*_max_ = 0.931	*l* = −19→17
9256 measured reflections	

### Refinement

**Table:**

Refinement on *F*^2^	Secondary atom site location: difference Fourier map
Least-squares matrix: full	Hydrogen site location: difmap and geom
*R*[*F*^2^ > 2σ(*F*^2^)] = 0.065	H atoms treated by a mixture of independent and constrained refinement
*wR*(*F*^2^) = 0.108	*w* = 1/[σ^2^(*F*_o_^2^) + 9.8897*P*] where *P* = (*F*_o_^2^ + 2*F*_c_^2^)/3
*S* = 1.13	(Δ/σ)_max_ < 0.001
1814 reflections	Δρ_max_ = 0.55 e Å^−^^3^
138 parameters	Δρ_min_ = −0.45 e Å^−^^3^
Primary atom site location: structure-invariant direct methods	Extinction correction: none

### Fractional atomic coordinates and isotropic or equivalent isotropic displacement parameters (Å^2^)

**Table:**

	*x*	*y*	*z*	*U*_iso_*/*U*_eq_	
Zn1	0.5000	0.0000	0.5000	0.0147 (2)	
O3	0.6625 (3)	0.0017 (4)	0.4416 (2)	0.0186 (7)	
H3A	0.6910	0.0850	0.4477	0.028*	
O2	0.5136 (3)	0.2237 (4)	0.5340 (2)	0.0179 (7)	
O1	0.6873 (3)	0.2905 (4)	0.4901 (2)	0.0229 (8)	
C3	0.3411 (5)	0.1656 (6)	0.2097 (4)	0.0290 (13)	
C2	0.4391 (5)	0.2285 (6)	0.2438 (4)	0.0254 (12)	
C5	0.3356 (4)	0.0065 (7)	0.3386 (3)	0.0206 (10)	
C6	0.5888 (4)	0.3201 (5)	0.5168 (3)	0.0172 (10)	
N1	0.4308 (3)	0.0673 (4)	0.3719 (3)	0.0156 (8)	
C1	0.4810 (4)	0.1762 (6)	0.3235 (3)	0.0199 (11)	
C7	0.5529 (4)	0.4835 (6)	0.5275 (4)	0.0224 (11)	
C4	0.2892 (5)	0.0528 (7)	0.2586 (4)	0.0279 (13)	
H4	0.224 (5)	0.010 (6)	0.243 (3)	0.025 (15)*	
H1	0.549 (4)	0.205 (6)	0.345 (3)	0.018 (13)*	
H3	0.308 (5)	0.199 (7)	0.154 (4)	0.035 (17)*	
H5	0.304 (4)	−0.066 (6)	0.376 (4)	0.026 (15)*	
H2	0.476 (5)	0.295 (6)	0.209 (4)	0.028 (16)*	
H7A	0.540 (4)	0.492 (6)	0.591 (4)	0.018 (13)*	
H7	0.616 (4)	0.549 (5)	0.514 (3)	0.014 (13)*	
H3B	0.694 (5)	−0.064 (7)	0.459 (4)	0.04 (2)*	

### Atomic displacement parameters (Å^2^)

**Table:**

	*U*^11^	*U*^22^	*U*^33^	*U*^12^	*U*^13^	*U*^23^
Zn1	0.0146 (3)	0.0125 (3)	0.0169 (4)	0.0005 (4)	0.0010 (3)	0.0002 (4)
O3	0.0190 (16)	0.0113 (16)	0.0254 (18)	0.0048 (18)	0.0012 (13)	0.0012 (17)
O2	0.0192 (16)	0.0116 (16)	0.0229 (16)	−0.0006 (14)	0.0033 (14)	0.0022 (14)
O1	0.0160 (15)	0.0137 (18)	0.039 (2)	−0.0010 (17)	0.0048 (15)	0.0000 (13)
C3	0.041 (3)	0.026 (3)	0.020 (3)	0.004 (2)	−0.012 (2)	0.001 (3)
C2	0.036 (3)	0.021 (3)	0.019 (3)	0.000 (2)	0.004 (2)	−0.006 (2)
C5	0.019 (2)	0.022 (3)	0.021 (2)	0.000 (3)	0.0002 (18)	0.001 (2)
C6	0.019 (2)	0.015 (2)	0.018 (3)	0.003 (2)	−0.0046 (18)	0.0011 (19)
N1	0.0181 (19)	0.012 (2)	0.017 (2)	0.0011 (17)	0.0012 (16)	0.0016 (17)
C1	0.020 (3)	0.018 (3)	0.022 (3)	−0.001 (2)	−0.002 (2)	−0.001 (2)
C7	0.020 (2)	0.014 (3)	0.034 (3)	−0.001 (2)	−0.005 (2)	−0.003 (2)
C4	0.028 (3)	0.026 (3)	0.029 (3)	0.003 (3)	−0.007 (2)	−0.005 (3)

### Geometric parameters (Å, °)

**Table:**

Zn1—O2	2.064 (3)	C2—C1	1.374 (7)
Zn1—O2^i^	2.064 (3)	C2—H2	0.90 (6)
Zn1—O3^i^	2.110 (3)	C5—N1	1.345 (6)
Zn1—O3	2.110 (3)	C5—C4	1.379 (7)
Zn1—N1^i^	2.170 (4)	C5—H5	0.93 (6)
Zn1—N1	2.170 (4)	C6—C7	1.525 (7)
O3—H3A	0.8200	N1—C1	1.348 (6)
O3—H3B	0.74 (6)	C1—H1	0.90 (5)
O2—C6	1.263 (6)	C7—C7^ii^	1.526 (9)
O1—C6	1.258 (5)	C7—H7A	0.97 (5)
C3—C2	1.383 (8)	C7—H7	0.97 (5)
C3—C4	1.387 (8)	C4—H4	0.89 (6)
C3—H3	0.96 (6)		
			
O2—Zn1—O2^i^	180.0	C1—C2—H2	123 (4)
O2—Zn1—O3^i^	88.60 (13)	C3—C2—H2	118 (4)
O2^i^—Zn1—O3^i^	91.40 (13)	N1—C5—C4	122.2 (5)
O2—Zn1—O3	91.40 (13)	N1—C5—H5	113 (3)
O2^i^—Zn1—O3	88.60 (13)	C4—C5—H5	125 (3)
O3^i^—Zn1—O3	180.0	O1—C6—O2	124.9 (4)
O2—Zn1—N1^i^	91.12 (14)	O1—C6—C7	119.4 (4)
O2^i^—Zn1—N1^i^	88.88 (14)	O2—C6—C7	115.7 (4)
O3^i^—Zn1—N1^i^	88.53 (14)	C5—N1—C1	117.3 (4)
O3—Zn1—N1^i^	91.47 (14)	C5—N1—Zn1	122.1 (3)
O2—Zn1—N1	88.88 (14)	C1—N1—Zn1	120.5 (3)
O2^i^—Zn1—N1	91.12 (14)	N1—C1—C2	123.5 (5)
O3^i^—Zn1—N1	91.47 (14)	N1—C1—H1	114 (3)
O3—Zn1—N1	88.53 (14)	C2—C1—H1	122 (3)
N1^i^—Zn1—N1	180	C6—C7—C7^ii^	110.8 (5)
Zn1—O3—H3A	109.5	C6—C7—H7A	103 (3)
Zn1—O3—H3B	108 (5)	C7^ii^—C7—H7A	112 (3)
H3A—O3—H3B	118.2	C6—C7—H7	110 (3)
C6—O2—Zn1	131.5 (3)	C7^ii^—C7—H7	114 (3)
C2—C3—C4	118.0 (5)	H7A—C7—H7	106 (4)
C2—C3—H3	122 (3)	C5—C4—C3	120.0 (5)
C4—C3—H3	120 (3)	C5—C4—H4	116 (3)
C1—C2—C3	118.9 (5)	C3—C4—H4	124 (4)
			
O3^i^—Zn1—O2—C6	−174.6 (4)	O3—Zn1—N1—C5	137.5 (4)
O3—Zn1—O2—C6	5.4 (4)	O2—Zn1—N1—C1	47.0 (4)
N1^i^—Zn1—O2—C6	96.9 (4)	O2^i^—Zn1—N1—C1	−133.0 (4)
N1—Zn1—O2—C6	−83.1 (4)	O3^i^—Zn1—N1—C1	135.6 (4)
C4—C3—C2—C1	0.5 (8)	O3—Zn1—N1—C1	−44.4 (4)
Zn1—O2—C6—O1	−16.8 (7)	C5—N1—C1—C2	1.0 (7)
Zn1—O2—C6—C7	161.3 (3)	Zn1—N1—C1—C2	−177.2 (4)
C4—C5—N1—C1	−0.5 (7)	C3—C2—C1—N1	−1.0 (8)
C4—C5—N1—Zn1	177.6 (4)	O1—C6—C7—C7^ii^	123.7 (6)
O2—Zn1—N1—C5	−131.1 (4)	O2—C6—C7—C7^ii^	−54.5 (7)
O2^i^—Zn1—N1—C5	48.9 (4)	N1—C5—C4—C3	0.1 (9)
O3^i^—Zn1—N1—C5	−42.5 (4)	C2—C3—C4—C5	−0.1 (9)

Symmetry codes: (i) −*x*+1, −*y*, −*z*+1; (ii) −*x*+1, −*y*+1, −*z*+1.

### Hydrogen-bond geometry (Å, °)

**Table:**

*D*—H···*A*	*D*—H	H···*A*	*D*···*A*	*D*—H···*A*
O3—H3A···O1	0.82	1.94	2.690 (5)	152
O3—H3B···O1^iii^	0.74 (6)	1.97 (6)	2.687 (5)	164 (5)

Symmetry codes: (iii) −*x*+3/2, *y*−1/2, *z*.
